# Metabolites of type I, II, III, and IV collagen may serve as markers of disease activity in axial spondyloarthritis

**DOI:** 10.1038/s41598-019-47502-z

**Published:** 2019-08-02

**Authors:** Markéta Hušáková, Anne-C. Bay-Jensen, Šárka Forejtová, Kateřina Zegzulková, Michal Tomčík, Monika Gregová, Kristýna Bubová, Jana Hořínková, Jindra Gatterová, Karel Pavelka, Anne Sofie Siebuhr

**Affiliations:** 1 Institute of Rheumatology and Department of Rheumatology, First Faculty of Medicine Charles University in Prague, Na Slupi 4, Prague, 128 50 Czech Republic; 2 Department of Rheumatology, Nordic Bioscience, Biomarkers and Research, Herlev, Denmark

**Keywords:** Ankylosing spondylitis, Predictive markers, Ankylosing spondylitis

## Abstract

Local inflammation in axial spondyloarthritis (axSpA) leads to the release of collagen metabolites from the disease-affected tissue. We investigated whether collagen metabolites were associated with disease activity and could distinguish non-radiographic(nr)-axSpA from ankylosing spondylitis (AS). A total of 193 axSpA patients (nr-axSpA, n = 121 and AS, n = 72) and asymptomatic controls (n = 100) were included. Serum levels of metalloproteinase (MMP)-degraded collagen type I (C1M), type II (C2M), type III (C3M) and type IV (C4M2) were quantified by enzyme-linked immunosorbent assay (ELISA). All metabolites were higher in axSpA than in controls (all p < 0.001). Serum levels of C1M, C3M, and C4M2 were increased in AS compared to nr-axSpA (43.4 ng/mL vs. 34.6; p < 0.001, 15.4 vs. 12.8; p = 0.001, and 27.8 vs. 22.4; p < 0.001). The best metabolite to differentiate between axSpA and controls was C3M (AUC 0.95; specificity 92.0, sensitivity 83.4). C1M correlated with ASDAS-CRP in nr-axSpA (ρ = 0.37; p < 0.001) and AS (ρ = 0.57; p < 0.001). C1M, C3M, and C4M2 were associated with ASDAS-CRP in AS and nr-axSpA after adjustment for age, gender, and disease duration. Serum levels of collagen metabolites were significantly higher in AS and nr-axSpA than in controls. Moreover, the present study indicates that collagen metabolites reflect disease activity and are useful biomarkers of axSpA.

## Introduction

Axial spondyloarthritis (axSpA) is a chronic inflammatory disorder that is characterised by sacroiliitis, inflammation of the spine and extra-musculoskeletal involvement. AxSpA generally manifests in early adulthood and men are more frequently affected than women. Most patients are also HLA-B27 positive. Two pathogenic events are the main contributors to disease burden, namely, disease-related inflammation and osteoproliferation in the axial skeleton^1^. Patients are divided into two sub-populations according to imaging features by the Assessment of SpondyloArthritis International Society (ASAS)^2^. Ankylosing spondylitis (AS) fulfils the modified New York criteria, including determination of radiographic sacroiliitis^3^, whereas the non-radiographic (nr)-axSpA does not meet these criteria. However, the inflammation-related pattern assessed by magnetic resonance imaging (MRI), such as bone marrow oedema with or without structural changes within the sacroiliac joints (SIJ), may be present in some nr-axSpA patients^4^. The clinical features of both axSpA subtypes include all, axial, peripheral and extra-articular symptoms. Back pain and peripheral arthritis or enthesitis are equally distributed between the axSpA subtypes, but uveitis occurs more frequently in AS^5^. Conventional synthetic disease-modifying drugs and biologics are currently recommended for the pharmacological treatment of the axSpA subtypes, but the monoclonal antibody tumour necrosis factor (TNF)-α inhibitor, infliximab and the inhibitor of interleukin (IL)-17 secukinumab are reserved only for AS^6^. Nr-axSpA was suggested as an early phase of AS because 4.9–11.6% of nr-axSpA patients develop radiographic sacroiliitis over two years^7,8^ and 19% develop radiographic sacroiliitis during a follow-up of 10 years^9^. Serological markers of disease activity that identify patients who structurally progress rapidly are highly needed for clinical trial enrichment and personalised health care^10,11^.

Inflammation is currently assessed by C-reactive protein (CRP), which is elevated in AS patients compared to nr-axSpA patients^8,12,13^. An increase in CRP may arise from many pathological events, such as a common cold or chronic inflammation. Therefore, CRP may not be able to identify the pathogenic-related inflammation occurring locally within the affected joints. The extracellular matrix (ECM) is a network of collagens, glycosaminoglycans and other molecules and fibrils filling the intercellular spaces that undergo substantial changes during inflammation and reflect pathological events, such as inflammatory cell influx. ECM proteins are prone to degradation by proteases, such as matrix metalloproteinases (MMPs), which result in the release of protease-specific metabolites. These MMP-degraded ECM metabolites that are produced locally during inflammatory escalated ECM turnover may be detected as serum biomarkers of ECM tissue turnover, which would reflect local pathogenic processes^14^. Serological levels of MMP-3, MMP-8, and MMP-9 are elevated in AS, especially in patients with higher disease activity and structural progression^15,16^.

Type I, II, III, and IV collagens are expressed in the ECM of different joint tissues (articular and hyaline cartilage, tendons, bone, and connective tissue) and are prone to degradation by proteases. The MMP-mediated metabolite of type I collagen (C1M) reflects soft tissue destruction, and it is elevated in AS^17^ and rheumatoid arthritis (RA)^18,19^. C2M is an MMP-mediated metabolite of type II collagen, and it reflects cartilage destruction^20^. An MMP-mediated metabolite of type III collagen (C3M) reflects soft tissue degradation. C2M and C3M are higher in AS patients compared to controls^17,21^, and C3M is found in the inflamed tissue of liver fibrosis^22^. The main collagen of the basement membrane is type IV collagen, and an MMP-mediated metabolite of type IV collagen (C4M2) is higher with soft tissue destruction^23^. C1M, C2M, and C3M were recently associated with the response to biologic therapy in AS^21^ and were correlated with CRP, erythrocyte sedimentation rate (ESR) and radiographic severity^24^. Therefore, higher ECM turnover might be a common pathogenic event in axSpA, and products of MMP-degraded collagens may be biomarkers of disease activity in axSpA. However, whether the levels of MMP-degraded collagen products are different between radiographic and non-radiographic axSpA and whether they are associated with disease activity is unknown.

In our study, we investigated the following factors: (1) the profile of serological MMP-mediated products of type I, II, III, and IV collagens (C1M, C2M, C3M and C4M2) in AS and nr-axSpA; (2) whether ECM metabolites could separate the two axSpA forms; and (3) whether these metabolites were associated with disease activity.

## Results

### Demographic description

The demographics of AS and nr-axSpA patients are characterised in Table 1. AS and nr-axSpA patients differed significantly in the following variables: disease duration since the first symptoms, CRP levels, and gender (all p < 0.001) and peripheral arthritis (p < 0.01). AS patients exhibited higher structural scores (modified Stoke ankylosing spondylitis spine score [mSASSS], p < 0.05, Spondyloarthritis research consortium of Canada [SPARCC] MRI and Berlin MRI, both p < 0.01) compared to nr-axSpA patients, but this difference was due to the group inclusion criteria. Both subgroups of axSpA were comparable in current or previous medications, disease activity and quality of life (Table 1 and Supplementary Table S1).

**Table 1:** Demographic and Biomarker’s Table of the study.

	nr-axSpA (n = 121)		AS (n = 72)		nr-axSpA vs. AS
	Mean	95% CI	Mean	95% CI	P-value
Age at the time of study (years)	37.5	35.5–39.6	34.5	32.3–36.8	*0*.*110*
Disease duration since first symptom (years)	7.9	6.3–9.5	10.0	7.9–12.1	<*0*.*001*
Gender (% female)	59		32		<*0*.*001*
BMI	25.1	24.2–26.0	24.4	23.5–25.4	*0*.*390*
ASDAS-CRP	2.0	1.8–2.2	2.2	2.0–2.4	*0*.*130*
ASQoL	5.3	4.4–6.2	5.7	4.5–6.8	*0*.*450*
BASDAI	3.0	2.6–3.4	2.6	2.1–3.0	*0*.*410*
BASFI^#^	1.0	0.7–1.4	1.3	1.0–1.9	*0*.*190*
EQ-5D	0.66	0.61–0.71	0.66	0.60–0.72	*0*.*400*
Swollen joint count	0.43	0.24–0.62	0.17	−0.014–0.35	*0*.*009*
Current therapy: NSA (%)	31		33		*0*.*749*
Current therapy: csDMARD/bDMARD (%)	27/1		11/4		*0*.*299/0*.*142*
Berlin MRI^#^	2.0	1.0–2.0	4.5	2.4–7.0	*0*.*003*
mSASSS	0.74	0.17–1.31	4.83	1.47–8.19	*0*.*018*
SPARCC MRI	6.8	5.2–8.5	14.7	10.2–19.3	*0*.*003*
***Biomarker’s levels***					
CRP (mg/l)^#^	2.7	1.7–4.0	7.7	4.4–12.0	<*0*.*001*
ESR (mm/h)	11.3	9.0–13.5	14.3	11.3–17.3	*0*.*073*
C1M (ng/ml)	34.6	29.3–36.5	43.4	38.0–51.1	<*0*.*001*
C2M (ng/ml)	0.36	0.34–0.38	0.35	0.32–0.37	*0*.*856*
C3M (ng/ml)	12.8	12.3–13.8	15.4	14.3–16.2	*0*.*001*
C4M2 (ng/ml)	22.4	21.0–24.8	27.8	25.4–30.8	<*0*.*001*

Mean or median with 95% CI - confidence interval is presented. Characteristics marked with # indicate where the median was used. Mann-Whitney T-test and Fisher’s exact test were used.

Abbreviations: AS: Ankylosing spondylitis, nr-axSpA: non-radiographic axial spondyloarthritis, BMI: body mass index, CRP: C-reactive protein, ESR: Erythrocyte sedimentation rate, ASDAS-CRP: AS disease activity score CRP, ASQoL: AS quality of life, BASDAI: Bath AS disease activity score, BASFI: Bath AS functional index, EQ-5D: EuroQol five dimension scale, NSA: non-steroidal antirheumatic drugs, csDMARD: conventional synthetic disease-modifying drugs, bDMARD: biological disease-modifying drugs, MRI: magnetic resonance imagining, mSASSS: modified Stoke ankylosing spondylitis spine score, SPARCC: Spondyloarthritis research consortium of Canada, C1M: metalloproteinase (MMP)-degraded type I collagen, C2M: MMP-degraded type II collagen, C3M: MMP-degraded type III collagen, C4M2: MMP-degraded type IV collagen alpha 3.

Asymptomatic controls were sex and age matched to nr-axSpA patients, but in comparison to AS were older (age at the time of analysis: 38.1; 95% CI 36.3–39.9), had different gender distribution (women predominance 51%) and higher body mass indexes (BMIs) (mean 35.4; 95% CI 24.7–26.1); all p < 0.05. As expected, both AS and nr-axSpA had significantly higher CRP levels than asymptomatic controls (mean 1.22; 95% CI 0.88–1.61), all p < 0.001.

### ECM metabolism is accelerated in axSpA patients particularly in the AS subgroup

MMP-degraded collagen type I, II, III and IV products were significantly increased in axSpA patients compared to asymptomatic controls (Supplementary Table S2). We looked for the relationships between all four biomarkers. C1M, C3M, and C4M2 were moderately to highly correlated to each other (all Spearman’s ρ > 0.53; all p < 0.001) and C2M was only weakly correlated to C1M (ρ = 0.25; p < 0.001), but not to the others biomarkers (Supplementary Table S3).

**Figure 1:**
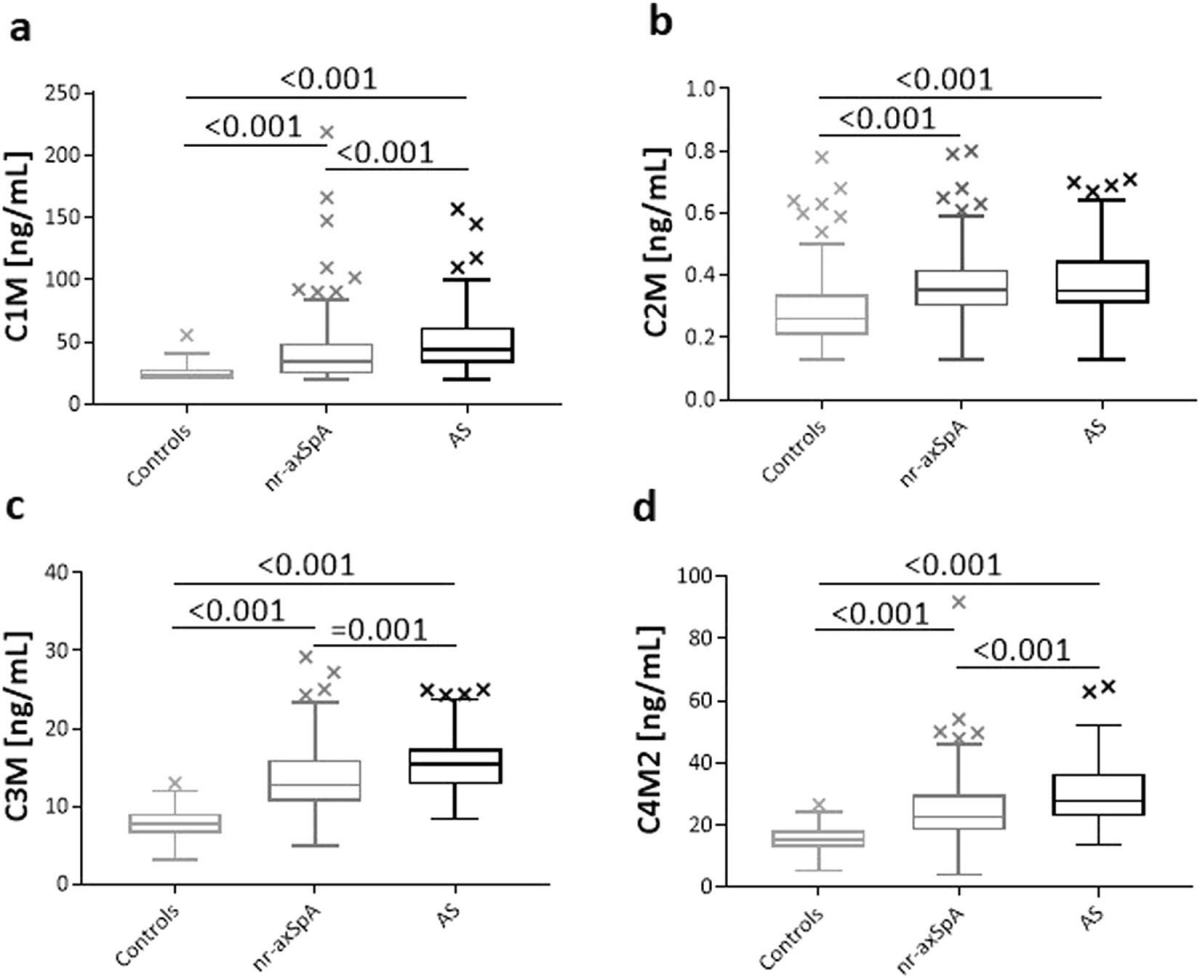
Metabolite levels in the AS group, nr-axSpA group and asymptomatic controls. (**a**) Type I collagen degraded by MMPs. (**b**) Type II collagen degraded by MMPs. (**c**) Type III collagen degraded by MMPs. (**d**) Type IV collagen degraded by MMPs. Kruskal-Wallis was used for differences between the three groups with Dunn’s multi-comparisons test. Statistical significant differences between groups are reported (P < 0.001 or P = 0.001). Data are shown as Tukey’s box plots.

Patients of both axSpA subtypes, namely, AS and nr-axSpA, expressed significantly higher levels of all four collagen metabolites than the asymptomatic controls: C1M 43.4 (95% CI 38.0–51.1) and 34.6 (29.3–36.5) vs. 24.5 (20.4–24.3) respectively; C2M 0.35 (0.32–0.37) and 0.36 (0.34–0.38) vs. 0.26 (0.24–0.28) respectively; C3M 15.4 (14.3–16.2) and 12.8 (12.3–13.8) vs. 7.8 (7.1–8.3), respectively; and C4M2 27.8 (25.4–30.8) and 22.4 (21.0–24.8) vs. 15.2 (14.7–15.7) respectively (Fig. 1a–d, all p < 0.001). However, the serum levels of the MMP-degraded products of collagen C1M, C3M, C4M2 were significantly higher in AS patients compared to nr-axSpA patients (p < 0.001, p = 0.001, p < 0.001, respectively), but the levels of C2M were comparable between AS and nr-axSpA patients (Fig. 1a–d, Table 1). In both subgroups, the three biomarkers: C1M, C3M and C4M2 correlated significantly to each other (for all analyses, Spearman’s ρ > 0.40; all p < 0.001), Table 2. The biomarker C2M, however, correlated only modestly with C1M (for AS Spearman’s ρ = 0.36; p < 0.01 and for nr-axSpA Spearman’s ρ = 0.19; p<0.05), but not with C3M and C4M2 (Table 2).

**Table 2:** Correlation of the MMP-degraded collagen type I, II, III and IV serum products with clinical variables and biomarkers.

	*C1M*				*C2M*				*C3M*				*C4M2*			
	nr-axSpA		AS		nr-axSpA		AS		nr-axSpA		AS		nr-axSpA		AS	
	ρ	*P value*	ρ	*P value*	ρ	*P value*	ρ	*P value*	ρ	*P value*	ρ	*P value*	ρ	*P value*	ρ	*P value*
Age at diagnosis	−0.05	*0*.*59*	0.17	*0*.*16*	−0.02	*0*.*79*	−0.08	*0*.*045*	0.03	*0*.*78*	0.19	*0*.*12*	−0.05	*0*.*57*	0.20	*0*.*099*
Disease duration	−0.03	*0*.*76*	0.12	*0*.*32*	−0.04	*0*.*57*	−0.02	*0*.*76*	−0.00	*0*.*99*	0.23	*0*.*057*	−0.11	*0*.*24*	0.14	*0*.*24*
SJC	0.10	*0*.*27*	0.06	*0*.*63*	0.21	*0*.*035*	0.16	*0*.*21*	−0.01	*0*.*90*	−0.03	*0*.*83*	0.03	*0*.*78*	0.12	*0*.*33*
CRP	**0**.**57**	**<*0***.***001***	**0**.**71**	**<*0***.***001***	0.12	*0*.*19*	0.21	*0*.*075*	**0**.**36**	**<*0***.***001***	**0**.**44**	**<*0***.***001***	**0**.**42**	**<*0***.***001***	**0**.**59**	**<*0***.***001***
ESR	**0**.**38**	**<*0***.***001***	**0**.**66**	**<*0***.***001***	0.01	*0*.*94*	**0**.**39**	**<*0***.***001***	**0**.**30**	**<*0***.***001***	**0**.**36**	***0***.***0019***	**0**.**35**	**<*0***.***001***	**0**.**53**	**<*0***.***001***
ASDAS-CRP	**0**.**37**	**<*0***.***001***	**0**.**57**	**<*0***.***001***	0.06	*0*.*62*	**0**.**34**	***0***.***016***	**0**.**27**	***0***.***0029***	0.28	*0*.*07*	0.24	*0*.*0079*	**0**.**38**	***0***.***010***
BASDAI	0.11	*0*.*23*	0.25	*0*.*038*	0.05	*0*.*83*	0.23	*0*.*13*	0.10	*0*.*30*	0.14	*0*.*023*	0.02	*0*.*86*	0.13	*0*.*28*
ASQoL	−0.05	*0*.*56*	**0**.**33**	**<*0***.***01***	0.02	*0*.*77*	**0**.**38**	***0***.***0046***	0.07	*0*.*42*	0.07	*0*.*58*	−0.11	*0*.*23*	0.17	*0*.*15*
EQ-5D	0.01	*0*.*88*	−0.31	*0*.*009*	−0.08	*0*.*59*	−0.32	*0*.*007*	−0.01	*0*.*93*	−0.15	*0*.*22*	0.11	*0*.*22*	−0.23	*0*.*052*
BASFI	0.01	*0*.*94*	**0**.**38**	**<*0***.***001***	−0.05	*0*.*41*	0.29	*0*.*016*	−0.04	*0*.*64*	0.20	*0*.*091*	−0.06	*0*.*55*	0.29	*0*.*012*
Berlin MRI	0.03	*0*.*80*	0.26	*0*.*18*	−0.11	*0*.*30*	0.32	*0*.*071*	−0.10	*0*.*35*	0.02	*0*.*95*	−0.06	*0*.*55*	0.11	*0*.*58*
mSASSS	−0.02	*0*.*87*	0.33	*0*.*009*	0.07	*0*.*52*	0.02	*0*.*95*	−0.08	*0*.*47*	0.23	*0*.*77*	−0.09	*0*.*42*	0.29	*0*.*026*
SPARCC MRI	0.06	*0*.*56*	0.29	*0*.*14*	−0.09	*0*.*41*	0.34	*0*.*075*	−0.09	*0*.*42*	0.02	*0*.*94*	−0.04	*0*.*72*	0.11	*0*.*58*
C1M	—	—	—	—	**0**.**19**	***0***.***034***	**0**.**36**	***0***.***002***	**0**.**52**	**<*0***.***001***	**0**.**40**	**<*0***.***001***	**0**.**61**	**<*0***.***001***	**0**.**58**	**<*0***.***001***
C2M	—	—	—	—	—	—	—	—	0.10	*0*.*28*	0.17	*0*.*16*	0.02	*0*.*87*	0.20	*0*.*087*
C3M	—	—	—	—	—	—	—	—	—	—	—	—	**0**.**71**	**<*0***.***001***	**0**.**63**	**<*0***.***001***

Spearman’s correlation between serological metabolites and clinical assessments were done. After Bonferroni correction, the alpha value was 0.00625 (0.05/8). Spearman’s rho (ρ) and p- values are presented. The italic P value demonstrates the exact value before correction, the bolt italic P value determines significance on p < 0.05; p < 0.01 and p < 0.001 after correction. The recurring values (ρ and P value) of the correlation between C1M, C2M, C3M and C4M2 are not shown in the Table.

Abbreviations: AS: Ankylosing spondylitis, nr-axSpA: non-radiographic axial spondyloarthritis, SJC: swollen joint count, CRP: C-reactive protein, ESR: Erythrocyte sedimentation rate, ASDAS-CRP: AS disease activity score CRP, BASDAI: Bath AS disease activity score, ASQoL: AS quality of life, EQ-5D: EuroQol five-dimension scale, BASFI: Bath AS functional index, MRI: magnetic resonance imagining, mSASSS: modified Stoke ankylosing spondylitis spine score, SPARCC: Spondyloarthritis research consortium of Canada, C1M: metalloproteinase (MMP)-degraded type I collagen, C2M: MMP-degraded type II collagen, C3M: MMP-degraded type III collagen, C4M2: MMP-degraded type IV collagen alpha 3.

### The products of ECM turnover differentiated AS and nr-axSpA from asymptomatic controls

While serum levels of all four tested MMP-degraded collagen products and CRP levels were higher in AS and nr-axSpA than in controls we investigated their abilities to identify axSpA patients (AS and nr-axSpA) from asymptomatic controls using Area under receiver operator characteristics curve (AUC ROC). C3M had the highest AUC to differentiate between axSpA and asymptomatic controls. The AUC of C3M was 0.95, with a specificity and sensitivity of 92.0 and 83.4, respectively, and an odds ratio of 30.9 (95% CI 4.0–236.7), see Table 3. C3M was also the best metabolite for the identification of nr-axSpA and AS patients from asymptomatic controls. The AUC for nr-axSpA was 0.93 [specificity 80.0 and sensitivity 92.6, odds ratio of 22.9 (3.0–175.7)], and the AUC for AS was 0.98 [specificity 92.0, sensitivity 91.7, odds ratio of 111.2 (37.7–327.7)], see Table 3. An AUC higher than 0.90 was also found for C1M and C4M2 to discriminate AS and asymptomatic controls, but with lower sensitivity and specificity.

**Table 3:** Diagnostic utility values of the collagen metabolite levels for axial spondyloarthritis.

*Biomarker*	*Criterion* (*cutt-off*)	*AUC*	*Specificity*	*Sensitivity*	*Odds ratio* (*95% CI*)	
**AS vs**. **asymptomatic controls**						
C1M	>29.0	0.90*	79.0	87.5	24.8	(10.7–57.7)
C2M	>0.28	0.71*	57.0	82.9	6.6	(3.2–13.8)
C3M	>10.5	0.98*	92.0	91.7	111.2	(37.7–327.7)
C4M2	>20.6	0.96*	91.0	87.5	70.8	(26.6–188.2)
CRP	>4.2	0.82*	88.0	62.5	10.2	(4.9–21.4)
**nr-axSpA vs**. **asymptomatic controls**						
C1M	>32.3	0.77*	90.0	54.5	9.1	(4.5–18.4)
C2M	>0.29	0.72*	61.0	78.2	5.2	(2.9–9.3)
C3M	>6.3	0.93*	80.0	92.6	22.9	(3.0–175.7)
C4M2	>16.9	0.84*	72.0	84.3	13.8	(7.2–26.6)
CRP	>2.3	0.66*	76.0	53.7	3.3	(1.9–5.9)
**axSpA vs**. **asymptomatic controls**						
C1M	>32.3	0.82*	90.0	62.7	12.6	(6.4–24.6)
C2M	>0.29	0.71*	61.0	77.2	5.3	(3.1–8.9)
C3M	>10.5	0.95*	92.0	83.4	30.9	(4.0–236.7)
C4M2	>20.5	0.89*	90.0	73.6	7.2	(4.2–12.3)
CRP	>3.8	0.72*	85.0	51.3	3.0	(1.8–5.1)
**AS vs**. **nr-axSpA**						
C1M	>29.2	0.65*	41.3	87.5	4.6	(2.1–10.1)
C2M	>0.42	0.51	76.0	31.9	1.4	(0.8–2.8)
C3M	>13.3	0.64*	57.0	73.6	7.6	(3.4–16.7)
C4M2	>23.3	0.67*	55.4	73.6	3.5	(1.8–6.5)
CRP	>10.7	0.66*	82.6	44.4	3.6	(1.9–6.9)

The analyses were performed using AUC ROC, and p-values, specificity and sensitivity are provided. Criterion (cut-off) determined the serum levels of each biomarker to differentiate between groups. *Means statistical significance of p < 0.001.

Abbreviations: AS: ankylosing spondylitis, nr-axSpA: non-radiographic axial spondyloarthritis, C1M: metalloproteinase (MMP)-degraded type I collagen, C2M: MMP-degraded type II collagen, C3M: MMP-degraded type III collagen, C4M2: MMP-degraded type IV collagen alpha 3, AUC: Area under receiver operator characteristics curve.

Moreover, for identifying AS or nr-axSpA from asymptomatic controls, all tested MMP-degraded collagen products, except C2M in the case of AS, had higher AUCs than CRP (see Table 3). The AUC of CRP levels was lowest of all serum biomarkers for the separation of nr-axSpA from asymptomatic controls (see Table 3). On the other hand, however, C1M, C3M, C4M2, and CRP had significant, albeit weak, AUCs for differentiating AS from nr-axSpA (AUC 0.65, 0.64, 0.67 and 0.66, respectively), Table 3.

We also investigated the difference in biomarker levels between groups when adjusted for age, gender, BMI, disease duration and CRP using logistic regression. The C1M, C3M and C4M2 remain significant after adjustment for age, gender, disease duration and BMI and only C4M2, when additionally adjusted for CRP (Supplementary material, Table S4).

**Table 4:** Multiple regression analyses of the relationship between MMP-degraded collagen type I, II, III and IV products and ASDAS-CRP.

		*Unadjusted*		*P value*	*Adjusted for age*, *gender*, *BMI*, *disease duration*		*P value*
		Beta (SD)	r-partial		Beta (SD)	r-partial	
*C1M*	**AS**	0.42 (0.10)	0.46	<*0*.*0001*	0.57 (0.11)	0.47	*0*.*0001*
	**nr-axSpA**	0.39 (0.07)	0.46	<*0*.*0001*	0.40 (0.07)	0.46	<*0*.*0001*
*C2M*	**AS**	0.31 (0.11)	0.32	*0*.*0063*	0.30 (0.12)	0.30	*0*.*015*
	**nr-axSpA**	0.03 (0.10)	0.03	*0*.*76*	0.04 (0.10)	0.04	*0*.*66*
*C3M*	**AS**	0.36 (0.13)	0.31	*0*.*0086*	0.43 (0.14)	0.35	*0*.*0041*
	**nr-axSpA**	0.37 (0.09)	0.33	*0*.*0002*	0.34 (0.10)	0.32	*0*.*0005*
*C4M2*	**AS**	0.42 (0.11)	0.42	*0*.*0003*	0.44 (0.12)	0.43	*0*.*0004*
	**nr-axSpA**	0.40 (0.08)	0.41	<*0*.*0001*	0.41 (0.08)	0.43	<*0*.*0001*

Beta with SD and r-partial are shown, and the analyses were performed on z-scores. The dependent variables were C1M, C3M, C3M and C4M2, and the independent variable was ASDAS-CRP. The following covariates were tested: Age gender, BMI and disease duration.

Abbreviations: AS: ankylosing spondylitis, nr-axSpA: non-radiographic axial spondyloarthritis, BMI: body mass index, SD: standard deviation, C1M: metalloproteinase (MMP)-degraded type I collagen, C2M: MMP-degraded type II collagen, C3M: MMP-degraded type III collagen, C4M2: MMP-degraded type IV collagen alpha 3.

The C1M levels were higher in females than in males (median 38.0 [95% CI 34.8–45.7] vs. 52.6 [42.6–63.55], p = 0.025) in AS, but not in nr-axSpA (32.7 [26.8–38.7] vs. 34.8 [28.8–39.0], p = 0.77; data not shown). C3M levels in nr-axSpA patients were higher in patients with the extra-articular manifestations than in patients without this symptom (15.0 [13.8–16.8] vs. 12.2 [11.0–12.7], p = 0.0003; data not shown). No differences in all MMP-degraded collagen type I, II, III and IV products serum levels were observed within the AS and nr-axSpA groups with different disease duration, involvement of peripheral joint, presence of uveitis, smoking status, current or previous pharmacological therapy, or HLA-B27 status (data not shown).

### The MMP-degraded collagen type I, II, III and IV products are related to disease activity of AS and nr-axSpA

We next investigated the correlation between ECM turnover metabolites and clinical assessments. Due to correction for multiple comparisons, the alpha value was 0.00625 for this analysis. The ECM metabolites were not correlated with age, disease duration, or presence of peripheral arthritis reflected in the swollen joints count (SJC) (Table 2). C1M, C3M, and C4M2 were moderately to strongly correlated to CRP (for all analyses, Spearman’s ρ > 0.36; all p < 0.001) and ESR (for all analyses, Spearman’s ρ > 0.30; all p < 0.01) in the AS and nr-axSpA groups (Table 2). C2M moderately correlated to ESR only in AS patients (ρ = 0.39; p < 0.001), Table 2.

The values of the Bath AS function index (BASFI) and the quality of life questionnaires [EuroQol five dimension scale (EQ-5D), and AS quality of life (ASQoL)] were similar in the AS and nr-axSpA patients, but the correlations differed between the groups. C1M in the AS patients correlated to the BASFI (ρ = 0.38; p < 0.001), and C1M and C2M correlated with the ASQoL (ρ = 0.33 and ρ = 0.38, respectively; both p < 0.01), Table 2. There were no correlations of ECM turnover metabolite levels to the scores of radiographic severities, albeit C1M and C4M2 tended to correlate to mSASSS in the AS subgroup, Table 2.

Although the AS disease activity score (ASDAS)-CRP level was not different between AS and nr-axSpA patients, there were differences in correlations to the MMP-degraded collagen products. The C1M levels correlated with the ASDAS-CRP in the nr-axSpA (ρ = 0.37; p < 0.001) and AS (ρ = 0.57; p < 0.001) subgroups (Table 2). C2M and C4M2 correlated to ASDAS-CRP only in the AS patients (ρ = 0.34; p < 0.05 and ρ = 0.38; p = 0.01, respectively), and C3M correlated weakly to ASDAS-CRP only in the nr-axSpA patients (ρ = 0.27; p < 0.01), Table 2. However, disease activity assessed by the Bath disease activity index (BASDAI) did not correlate to the four products of collagen degradation. The differences in these results may be the correlation of the metabolites to CRP, which is part of the ASDAS-CRP score, but not the BASDAI.

The multiple regression model was used to investigate the relationship of MMP-degraded collagen products to disease activity (ASDAS-CRP) with adjustment for confounders. Notably, when metabolites were adjusted for age, gender, BMI, and disease duration, the relationship of all four biomarkers to ASDAS-CRP remained at the same level as the unadjusted model (Table 4). C2M reflected the disease activity only in AS. C1M, C3M and C4M2 reflected the changes of disease activity in both subgroups, but somewhat more clearly in AS. For example, if ASDAS-CRP was altered one unit, then C1M showed a change of 0.57 in AS and 0.40 in nr-axSpA patients (Table 4). However, for these analyses, the effect-size (beta) was too low, and the association was not considered clinically relevant.

## Discussion

This cross-sectional study investigated the level of ECM tissue turnover metabolites (C1M, C2M, C3M, and C4M2) in axSpA. We investigated whether MMP-degraded collagen metabolite levels, measured in serum, were different in radiographic axSpA (AS) compared to non-radiographic axSpA, and whether these metabolites were associated with disease activity. We found that the metabolite levels were higher in axSpA (both nr-axSpA and AS) compared to asymptomatic controls and all metabolites, except C2M, were higher in AS compared to nr-axSpA. Next, the ECM metabolites, C3M in particular, differentiated axSpA from asymptomatic controls, but not AS from nr-axSpA. Finally, C1M, C3M, and C4M2 were associated with quality of life, function index, and disease activity on the ASDAS-CRP in nr-axSpA and AS patients. All the tested biomarkers, except C2M in nr-axSpA patients, had weak to moderate associations to ASDAS-CRP. Our results indicate that collagen degradation metabolites are higher in axSpA and these metabolites may be disease activity biomarkers of axSpA.

Although numerous similarities, such as disease activity and response to pharmacological therapy, including TNF-α inhibitors, between nr-axSpA and AS were observed in longitudinal studies^5,8,13,25^, both forms of axSpA generally differ in the severity of inflammatory changes evaluated in serum using CRP assessment or imaging methods within the sacroiliac joints and spine^25,26^. As expected, features, such as predominance in women, shorter disease duration, milder radiographic status and lower CRP level, were more common in nr-axSpA than AS in our study. However, a higher occurrence of peripheral arthritis was found in the nr-axSpA subgroup compared to the AS subgroup. A recent meta-analysis revealed that the occurrence of peripheral arthritis tended to be higher in nr-axSpA than in AS^5^. More recently, de Winter *et al*.^27^ suggested that half of axSpA patients suffered from a combination of axial and peripheral symptoms, but these patients did not differ in the presence of radiographic sacroiliitis from patients with only pure axial symptoms.

All of the MMP-degraded collagen products assessed in this study were higher in nr-axSpA and AS patients than asymptomatic controls and correlated with CRP. All of the analysed products indicate an accelerated ECM turnover and reflect different events in the immune-musculoskeletal pathology of axSpA because collagen types I and III are expressed in the bone, tendons and ligaments, collagen type II is primarily expressed in the entheses and cartilages, and type IV is part of the basement membrane. All these collagens are naturally substrates for several metalloproteinases, including the MMPs that are highly expressed during inflammation. Serum levels of MMP-1, 2, 3, 8 and -9 reflect disease activity and progression^15,16,28^ and MMP-3 expressed locally within in the inflamed tissue^29^ participates in the activation of other MMPs and the changes that lead to osteogenesis^29^. The sources of MMPs may be fibroblasts and immune cells, such as macrophages. The serum levels of C1M, C3M and C4M2 in our study indicated an accelerated ECM turnover of soft tissue and joint structures. All three of these biomarkers tightly correlated with CRP and were higher in AS patients than in nr-axSpA patients, which may reflect the different degrees of inflammation of these two entities. An increased number of inflammatory spinal lesions in AS patients than in nr-axSpA patients was found previously^26,30^ and a recent study demonstrated increased entheseal abnormalities in axSpA patients with worse structural damage^31^. We did not find correlations between the severity of MRI lesions in the SIJ and biomarkers levels, but the mSASSS tended to correlate with C1M and C4M2 in AS. A recent study did not confirm C1M as a prognostic marker for the structural progression for AS patients with longstanding disease^32^. Bay-Jensen *et al*.^24^ demonstrated a correlation between C3M serum levels and disease activity and structural damage characterised by mSASSS in AS patients, and C3M showed a prognostic capacity for disease structural burden. However, the disease duration of our patients with AS and the scores of mSASSS were lower than in previous studies. Therefore, further work with axSpA patients at follow-up evaluations to monitor structural changes in disease is necessary to determine if the MMP-degraded collagen products may act as prognostic biomarkers, particularly at different stages of the disease. On the other hand, we detected increased levels of C2M in nr-axSpA and AS patients. The C2M biomarker was similar in both groups and it did not correlate with CRP or radiographic scores. This finding is consistent with Bay-Jensen’s study in which C2M was not associated with mSASSS or CRP in AS patients with longstanding disease, but high serum levels of C2M together with C3M predicted structural progression^24^. The main tissues with collagen type II are cartilage and entheses and our findings suggest similar pathogenetic events in the extracellular matrix in AS and nr-axSpA. However, a follow-up of nr-axSpA is necessary to determine whether C2M will have additional effects on the prognostic capacity of the other MMP-degraded collagen biomarkers. C1M and C3M were recently associated with disease activity in psoriatic arthritis^33^, and C1M serves as a metabolite of rapid structural progression in RA^18^. Kang *et al*.^34^ found that another serological type I collagen degradation metabolite, the C-terminal telopeptide of type I collagen (sCTX-I), reflected the intensity of MRI-established bone marrow oedema within the SIJ in AS, but not nr-axSpA, patients. In a SpA model, a lowered resistance to mechanical stress maintained the entheseal inflammation, which may induce new bone formation^35^. Increased degradation of type I, II, and IV collagens (C1M, C2M, C4M2) may result in the insufficient formation of the structures of the joints, enthesis, and adjacent structures and participate in their biomechanical insufficiency.

The overlap between AS and nr-axSpA may be associated with disease activity because patients with higher biomarker levels may be the patients who progress rapidly, and patients with lower levels may be stable patients. Although, the positive correlation between ASDAS-CRP and biomarkers in AS and nr-axSpA patients are supportive, this hypothesis should be tested in longitudinal studies. A recent work demonstrated the efficacy of C1M to reflect improvement of disease activity after TNF-α inhibitor therapy in AS patients^21^. The weak association between C2M and ASDAS-CRP in AS patients, but not in nr-axSpA patients, may reflect the severity of cartilage destruction in the fully blunted disease. This association indicates a relationship between disease activity and disease burden, but this connection must be confirmed. Based on previous results from AS and other pathologies, this type of biomarker could aid in the identification of patients with rapid progression, who are most in need of immediate treatment.

Although the mean ECM metabolites levels were higher in AS compared to nr-axSpA patients, there was too great of an overlap (large variation) in metabolite levels for the metabolites to provide good separation of the axSpA groups. C3M was the best metabolite for segregating AS from nr-axSpA and asymptomatic controls, which is consistent with a previous study that found that C3M was better than C2M^17^. C1M, C3M and C4M2 performed better in differentiating AS and nr-axSpA from asymptomatic controls than CRP. However, for true diagnostic purposes, the metabolites should be included in a diagnostic panel to ensure good specificity and sensitivity in the diagnosis and identification of disease subgroups. In our study, C3M has very high sensitivity for AS and nr-axSpA to discriminate from healthy individuals (91.7 and 92.6, respectively), but the specificity remain higher in AS (92.0) than in nr-axSpA (80.0). As the commonly used CRP has the lower specificity and sensitivity for AS (88.0 and 62.5, respectively) and nr-axSpA (76.0 and 53.7, respectively) in our study, the findings of C3M might suggest the new potential serological biomarker for both axSpA subtypes. Although these results show potential, larger studies are essential for the application of C3M as potential diagnostic choice for AS and nr-axSpA particularly if C3M will accomplish enough strength for prediction of the disease. However, whether a serological diagnostic criteria or method is needed is debatable, because these biomarkers would be assessed alongside the already used CRP. On the other hand, additional information about the C3M (and others MMP-degraded metabolites) in relation to disease phenotype and pathogenesis could be useful for therapeutic decisions in individual patient.

Axial spondyloarthritis is a longstanding disorder, and epidemiological studies suggest an increased cardiovascular disease risk^36^. Systemic cardiovascular impairment, such as atherosclerotic changes, were found in AS patients, but not nr-axSpA patients, but the number of atherosclerotic plaques was increased with extra-articular manifestations in nr-axSpA patients^37^. A large epidemiologic cohort of postmenopausal women found higher levels of C1M as an independent mortality risk factor for several diseases, including cancer and cardiovascular disorders^38^. Degradation of type I collagen assessed with C1M was higher in women with AS and nr-axSpA in the current study. The cardiovascular complications are estimated as most frequent in men with AS compared to the general population^39^, but an equal increased risk of cancer is estimated for both sexes^40^. Data in a recent meta-analysis showed that nr-axSpA and AS patients do not differ in extra-articular manifestations^5^, which was confirmed in the current study. Notably, C3M levels were higher in nr-axSpA patients with a prevalence of extra-articular manifestation compared to nr-axSpA patients with no extra-articular manifestation. This result illustrates that C3M is not exclusively originating from tissues of the joint but may originate from the tissues of the extra-articular manifestations. C1M and C3M reflect fibro-proliferative and cardiovascular changes, and future studies should elucidate the relationships between systemic involvement and collagen tissue turnover in axSpA. Understanding this relationship may be useful to distinguish patients with a higher risk for systemic complications for earlier and personalised treatment of these patients and provide knowledge of metabolites levels in patients with several pathologies. Each pathology may add to the total pool of metabolites and be a false positive for another pathology, if the pathology is undiagnosed.

Some limitations should be considered during the interpretation of the current work. First, this study was a cross-sectional study, and we were not able to investigate changes in biomarker levels with changes in disease activity over time. However, the disease duration since the first symptoms was long, and the disease may be well established with stable disease activity. Furthermore, the disease duration was significantly longer in AS patients compared to nr-axSpA patients. However, this difference was tested in the multiple regression analysis, and adjustment for disease duration did not change the results significantly. Second, another limitation is the lack of clinical information about cardiovascular and other systemic complications. These complications may affect the MMP-degraded collagen products levels and undermine the signals related to axSpA. Finally, our control group was asymptomatic individuals without personally reported clinical symptoms of back pain, but they did not undergo clinical or radiographic examination. Therefore, any underlying non-diagnosed diseases could have influenced the metabolite levels.

## Conclusion

This study demonstrated elevated serum levels of ECM metabolites (C1M, C2M, C3M, and C4M2) in radiographically determined forms of axSpA compared to controls. Our results support the distinct tissue degenerative events in different axSpA population (i.e. nr-axSpA and AS). The serum levels of products of MMP-degraded collagen, especially C1M, C3M and C4M2, was significantly associated with disease activity, linking tissue turnover with clinical activity of disease. C3M was the best biomarker at separating AS and nr-axSpA, but investigation of C3M as a true diagnostic tool within axSpA, must be studied in more and larger studies. However, this current study illustrates the potential of serological metabolites of tissue destruction as novel disease activity biomarkers in axSpA.

## Methods

### Patients

Patients (n = 193) with recently diagnosed axSpA were included into the Prague Axial SPondyloArthritis Cohort (PRASPAC)^41^. The inclusion criteria were a maximum of three years since the diagnosis of axSpA, characteristics of the first symptoms, and the disease course of axSpA.

The following set of clinical assessments were available for all patients: personal and family history; current and previous therapy; smoking history; BMI; clinical manifestation of axSpA; clinical determination of peripheral arthritis with SJC; evaluation enthesis involvement using the Maastrich Enthesitis Score (MASES)^2^; disease activity according to the ASDAS-CRP^42^; the BASDAI^43^; and the BASFI^43^. Patient-reported outcomes evaluating the quality of life, the ASQoL, and the EQ-5D^44,45^ were included. Imaging examinations consisted of X-rays of the SIJ and spine evaluated by two independent radiologists and one rheumatologist trained in the evaluation of X-rays in axSpA. Patients with radiographic sacroiliitis, according to the modified New York criteria^3^, were characterised as AS (n = 72). MRI of the SIJ was performed in cases of negative findings of the SIJ on X-ray and analysed according the ASAS^2^ independently by one radiologist and one rheumatologist with training in MRI assessment of axSpA. Patients without radiographic changes in the SIJ, but who fulfilled the ASAS criteria of MRI findings or HLA-B27 positivity together with clinical findings, were classified as nr-axSpA (n = 121). The following radiologic scoring systems were used to establish the severity of MRI or X-ray findings of the SIJ or spine: the SPARCC^46^ and the Berlin MRI grading system^47^ or the mSASSS^48^. Fasting blood samples (serum) for metabolite evaluation were collected from all patients at the first visit to the PRASPAC and stored at −70 °C until assayed. However, all patients had assessments of CRP and ESR at the time of blood draw.

One hundred asymptomatic individuals without any autoimmune or other inflammatory disorder, current infection, or surgery were used as a reference group for metabolite analyses.

All patients signed informed consent for inclusion into the clinical and laboratory database. The local Ethical Committee of the Institute of Rheumatology in Prague approved the consent form (reference number 959/2014 and 960/2014), and the Scientific Board of the Institute of Rheumatology in Prague authorised the study design and the database creation. The study was performed in compliance with the Declaration of Helsinki.

### Products of MMP-degraded collagen assessments

A panel of MMP-mediated products of collagen was measured in fasting serum using validated enzyme linked immunosorbent assays (ELISAs); the collagens types measured were MMP-degraded types I (C1M)^19^, II (C2M)^20^, III (C3M)^49^, and type IV collagen alpha 3 (C4M2)^50^ (Nordic Bioscience, Herlev, Denmark). All analyses were quality controlled with 2 kit controls and 3 in house quality controls. The inter- and intra-assay variations were below 15% and 10%, respectively. An acceptable linearity of <20% was observed, and no interference of biotin, haemoglobin or intralipid 20 was found. Sample measurements were accepted if the standard curve had an acceptable recovery of <20% in 75% individual standard curve assessments within the measurement range, and if 3 of the 5 control samples were accepted.

### Statistics

The summary statistics are shown in Table 1. Fisher’s exact test was used to identify differences in binary variables, and the Mann-Whitney U-test examined differences between levels in continuous variables because some assessments were not normally distributed. Kruskal-Wallis with Dunn’s multiple comparisons test was used for examining differences between product levels between the three groups (AS, nr-axSpA, and controls). Spearman’s correlation test was used for correlation analysis between the products and clinical assessments. Multiple regression and logistic regression analyses were used to test for correlations with adjustments for age, gender, BMI, and disease duration. Data for multiple regression were standardised by z-scores. An area under the receiver operating characteristics curve (AUC ROC) was used for examining the separation potential of the metabolites. Odds ratios were calculated from the cut-off values identified in the AUC ROC. Data analyses were performed using MedCalc Statistical Software version 17.6 (MedCalc Software bvba, Ostend, Belgium; http://www.medcalc.org; 2017). Graphical illustrations were created using GraphPad Prism version 6.00 for Windows (GraphPad Software, La Jolla California USA, www.graphpad.com).

## Supplementary information

Dataset 1

## Data Availability

The datasets generated during and/or analysed during the current study are available from the corresponding author on reasonable request.
