# Publisher Correction: Metabolites of type I, II, III, and IV collagen may serve as markers of disease activity in axial spondyloarthritis

**DOI:** 10.1038/s41598-020-71093-9

**Published:** 2020-08-19

**Authors:** Markéta Hušáková, Anne-C. Bay-Jensen, Šárka Forejtová, Kateřina Zegzulková, Michal Tomčík, Monika Gregová, Kristýna Bubová, Jana Hořínková, Jindra Gatterová, Karel Pavelka, Anne Sofie Siebuhr

**Affiliations:** 1 Institute of Rheumatology and Department of Rheumatology, First Faculty of Medicine, Charles University in Prague, Na Slupi 4, Prague, 128 50 Czech Republic; 2 Department of Rheumatology, Nordic Bioscience, Biomarkers and Research, Herlev, Denmark

Correction to: *Scientific Reports* 10.1038/s41598-019-47502-z, published online 02 August 2019

In the original version of this Article, Monika Gregová, Kristýna Bubová and Jana Hořínková were incorrectly affiliated with ‘Department of Rheumatology, Nordic Bioscience, Biomarkers and Research, Herlev, Denmark’. The correct affiliation is listed below.

Institute of Rheumatology and Department of Rheumatology, First Faculty of Medicine Charles University in Prague, Na Slupi 4, Prague, 128 50, Czech Republic

This error has now been corrected in the PDF and HTML versions of the Article.

